# Anatomical and Functional Deficits in Patients with Amnestic Mild Cognitive Impairment

**DOI:** 10.1371/journal.pone.0028664

**Published:** 2012-02-03

**Authors:** Ying Han, Su Lui, Weihong Kuang, Qi Lang, Ling Zou, Jianping Jia

**Affiliations:** 1 Department of Neurology, Capital Medical Universiy, Xuanwu Hospital, Beijing, China; 2 Department of Radiology, West China Hospital of Sichuan University, Chengdu, China; 3 Department of Psychiatry, West China Hospital of Sichuan University, Chengdu, China; 4 Department of Neurology, West China Hospital of Sichuan University, Chengdu, China; Institut d'Alta Tecnologia-PRBB, Spain

## Abstract

**Background:**

Anatomical and functional deficits have been studied in patients with amnestic mild cognitive impairment (MCI). However, it is unclear whether and how the anatomical deficits are related to the functional alterations. Present study aims to characterize the association between anatomical and functional deficits in MCI patients.

**Methods:**

Seventeen amnestic MCI patients and 18 healthy aging controls were scanned using a T1 Weighted MPRAGE sequence and a gradient-echo echo-planar imaging sequence. Clinical severity of MCI patients was evaluated by using Clinical Dementia Rating, Mini Mental State Examination (MMSE), Clock Drawing Test, Auditory Verbal Learning Test and Activities of Daily Living. VBM with DARTEL was used to characterize the gray matter deficits in MCI. Regional amplitude of low-frequency (0.01–0.08 Hz) fluctuations (ALFF) was used to evaluate regional functional alteration in MCI and fractional ALFF(fALFF) in slow 4 (0.027–0.073 Hz) and slow 5 (0.01–0.027 Hz) were also calculated.

**Results:**

Significantly decreased gray matter volume (GMV) was observed in amnestic MCI group mainly in bilateral prefrontal, left temporal and posterior cingulate cortex. Significant positive correlation was observed between the GMV in left inferior frontal gyrus and MMSE scores. Interestingly, decreased ALFF/fALFF was revealed in MCI group compared to controls mainly in prefrontal, left parietal regions and right fusiform gyrus, while the increased ALFF/fALFF was found in limbic and midbrain. Furthermore, the changes of fALFF in MCI in the slow-5 band were greater than those in the slow-4. No significant correlation was found between the morphometric and functional results.

**Conclusions:**

Findings from the study document that wide spread brain volume reduction accompanied with decreased and increased regional function in MCI, while the anatomical and functional changes were independently. Therefore, the combination of structural and functional MRI methods would provide complementary information and together advance our understanding of the pathophysiology underlying the symptoms of MCI.

## Introduction 

Mild cognitive impairment (MCI) is always being recognized as a transitional state between normal aging and dementia, and revealing the cerebral deficits of MCI would help neurologists in earlier detection and treatment of dementia [1]. Although past morphometry and functional MRI studies [2]–[6] have revealed independent anatomical and functional deficits mainly in frontal, temporal and limbic regions in patients with MCI, one important issue arises recently as to whether and how the anatomical deficits are related to the functional alterations. In patients with dementia, a strict relationship between hypometabolism and gray matter atrophy was observed [7]. However, inconsistent distributions between structural [4], [5], [8]–[10] and metabolic changes [11], [12] have been found in patients with MCI by independent studies of anatomy and function. Only a few studies used anatomical and functional methods in combination in MCI, and the results were inconsistent. A previous study [13] of 21 patients with MCI revealed dissociation of gray matter atrophy and hypometabolism by combination of MRI and positron emission tomography (PET), while our recent resting state fMRI study reported both increased and decreased regional function, which were not significantly influenced by the gray matter loss in the MCI patients [14]. Two recent studies combined anatomic MRI and resting state fMRI in MCI patients, and demonstrated dissociation of gray matter atrophy and altered functional connectivity [4], [8]. These studies implicated that deficits of MCI arise from systems-level disturbances of both anatomy and function and there is a complicate relationship between the deficits of cerebral anatomy and functional alteration.

In recent years, voxel based morphometry (VBM) is becoming a spatially specific and unbiased method for exploring regional gray matter volume which has already been successfully applied to schizophrenia[15], [16], dementia [5] and MCI [3]. For functional analysis, “resting state” fMRI not only avoids performance confounds [17] but is easier to implement in the context of clinical studies to assess resting state brain physiology than PET/Single-photon emission computed tomography (SPECT), due to its lower cost, greater availability and non-invasiveness [18]. Spontaneous low-frequency blood-oxygen-level-dependent (BOLD) fluctuations have been observed by fMRI during the “resting” state in both human and animal models. These signals bear numerous similarities to fluctuations in neurophysiological, hemodynamic and metabolic parameters [19]. Both animal [20] and human studies [21], [22] indicated that the regional amplitude of low-frequency fluctuations (0.01–0.08 Hz; ALFF) reflects spontaneous synchronous neural activity during resting state fMRI studies. Such metric was found reliable and could be used to characterize the regional functional changes in patients [23]. Two kinds of parameters could be used to evaluate the regional amplitude of low-frequency fluctuations, i.e., ALFF and fractional ALFF(fALFF). ALFF measures the total power of a given time course within a specific frequency range [24], and fALFF measures the power within a specific frequency range divided by the total power in the entire detectable frequency range [25]. Han et al. [14] had revealed that the changes of ALFF/fALFF in MCI in the slow-5 band (0.01–0.027 Hz) were greater than those in the slow-4 (0.027–0.073 Hz) which suggest the pattern of intrinsic brain activity of MCI is sensitive to specific frequency bands.

Thus, the present study was designed to 1) identify regional gray matter deficits and alteration of ALFF/fALFF in drug-naïve patients with MCI; and 2) to investigate the relationship between the deficits of gray matter volume and functional alteration, as well as their association with clinical symptoms.

## Results

### Morphometry study

**Figure 1:**
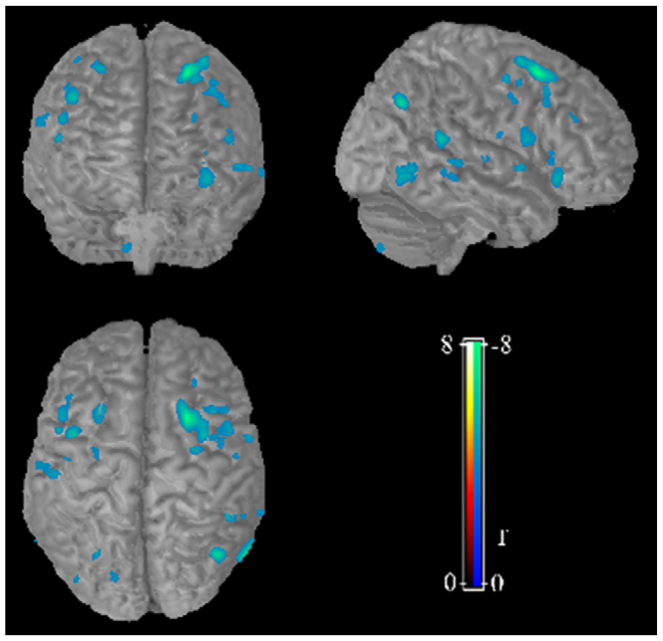
VBM analysis revealed decreased gray matter volume in MCI group compared to controls. Compared to the control group, the MCI group showed significantly decreased GMV (blue region) in mainly in bilateral prefrontal, left temporal and posterior cingulate cortex.

Compared to the control group, the MCI group showed significantly decreased GMV mainly in bilateral prefrontal, left temporal and posterior cingulate cortex (Table 1, Figure 1). Left middle frontal gyrus survived even after family wised error correction (p = 0.047, corrected with FDR). No significant increase in GMV was found in the MCI group compared to healthy controls. For validating the GMV results, the voxel-wise reproducibility map was generated (Information S1). Overall, our findings exhibited high reproducibility (p<0.01, degree of freedom = 30). Specifically, left inferior frontal gyrus demonstrated the highest reproducibility across validation analyses.

**Table 1:** VBM analysis revealed decreased gray matter volume in MCI group compared to controls.

Point of maximal change	Side	Talairach(mm)			Voxel size	P-corrected with FDR	t-score
		X	Y	Z			
Middle frontal gyrus	L	−25	16	47	982	0.047*	5.8
Angular gyrus	L	−44	−62	35	292	0.058	5.0
Inferior Temporal gyrus	L	−61	62	−3	424	0.051	4.7
Superior Temporal gyrus	L	−50	−41	15	292	0.067	4.5
Precentral gyrus	R	41	7	35	185	0.077	4.4
Inferior frontal gyrus	L	−37	24	−9	179	0.096	4.0
Posterior cingulate cortex	R	12	−51	24	43	0.097	3.9
Precentral gyrus	R	48	7	13	25	0.098	3.9
Superior frontal gyrus	R	24	20	50	117	0.098	3.8
Middle frontal gyrus	L	−30	−3	45	54	0.099	3.8
Inferior frontal gyrus	R	46	15	23	90	0.099	3.8
Precentral gyrus	L	−50	7	13	104	0.099	3.8

*: p<0.05, corrected at whole brain level;

**: p<0.05, after small volume correction (SVC).

Significant positive correlation was observed between the GMV in left inferior frontal gyrus and MMSE scores (r = 0.81, p = 0.026). No significant correlation was found between the GMV of the other regions and the clinical symptom severity.

### ALFF study

**Figure 2:**
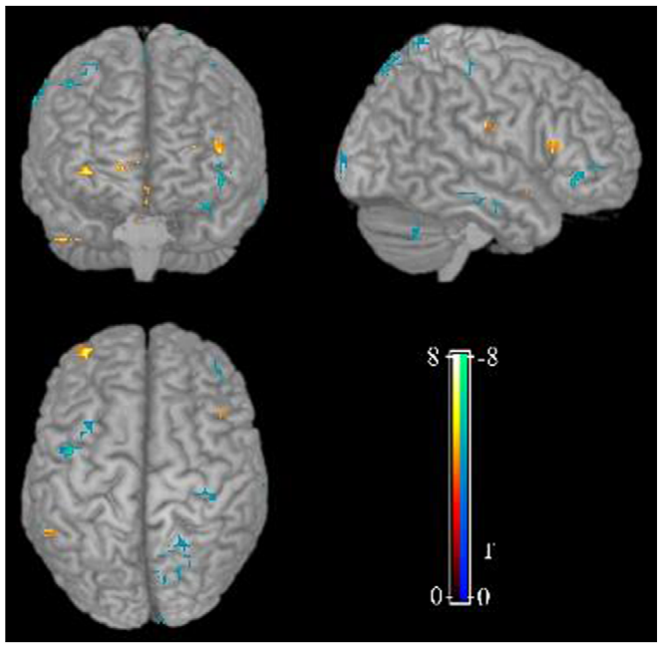
ALFF analysis revealed regional functional alteration in MCI group compared to controls. Compared to the control group, both increased and decreased regional function was revealed in MCI group in relative to controls.

Interestingly, both increased and decreased regional function was revealed in MCI group in relative to controls (Figure 2). The decreased ALFF were observed mainly in bilateral prefrontal, left parietal regions and right fusiform gyrus, while the increased ALFF were mainly found in limbic regions and midbrain (Table 2). ALFF analysis for slow 4 [0.027–0.073 Hz] and slow 5 [0.01–0.027 Hz] band also revealed decreased ALFF in cingulate cortex, prefrontal cortex, occipital regions and the bilateral parietal regions, while increased ALFF was observed in superior temporal gyrus, limbic regions and midbrain (Information S1). Furthermore, the changes of ALFF in MCI in the slow-5 band were greater than those in the slow-4. However, such alteration did not correlated with any of the clinical symptom severity.

**Table 2:** ALFF analysis revealed both increased and decreased regional function in MCI group compared to controls.

Point of maximal change	Side	Talairach(mm)			Voxel size	*P*-corrected with FDR	t-score
		X	Y	Z			
**MCI<controls**							
Inferior frontal gyrus	L	−44	31.00	−9	13	0.012	4.2
Supramarginal gyrus	R	62	−45	38	22	0.002	4.1
Superior parietal lobule	L	−21	−64	64	26	0.001	3.9
Fusiform gyrus	R	36	−53	−17	12	0.015	3.6
Postcentral gyrus	L	−18	−46	71	11	0.02	3.2
Middle frontal gyrus	R	33	14	54	10	0.026	3.2
**MCI>controls**							
Insula	R	27	−28	26	15	0.008	4.6
Superior temporal gyrus	R	50	−34	10	16	0.007	3.9
Midbrain	R	6	−24	−4	12	0.015	3.8
Midbrain	L	−3	−21	−14	10	0.026	3.8
Parahippocampal gyrus	R	21	−44	−13	12	0.016	3.7
Anterior cingulate	R&L	12	38	−2	15	0.008	3.5

Global ALFF for slow 4 and slow 5 between MCI and controls were not significant (p = 0.34 for slow 4, p = 0.12 for slow 5). Leave-one-out reproducibility analysis were also did for all above ALFF analysis and all results showed high reproducibility (p<0.01, degree of freedom = 25), especially for left superior parietal lobule which demonstrated the highest reproducibility across validation analyses (Information S1).

### fALFF study

We compared the fALFF between MCI and controls and found that the MCI group showed wide spread decreased fALFF mainly in bilateral medial/dorsallateral prefrontal cortex, cingulate gyrus, occipital regions and left cerebellum. Increased fALFF was revealed only in brain stem regions. Compared with ALFF findings, fALFF revealed more regions with decreased activity, while less regions with increased activity (Information S1). fALFF analysis for slow 4 [0.027–0.073 Hz] and slow 5 [0.01–0.027 Hz] band also revealed decreased fALFF in cingulate cortex, prefrontal cortex, occipital regions and the bilateral parietal regions, while increased fALFF was observed in superior temporal gyrus, limbic regions and midbrain (Information S1). Furthermore, the changes of fALFF in MCI in the slow-5 band were greater than those in the slow-4 (Information S1). However, no overlap nor inter-subject correlation was observed between the fALFF and GMV alternations. Global fALFF for slow 4 and slow 5 between MCI and controls were not significant (p = 0.44 for slow 4, p = 0.27 for slow 5). Leave-one-out reproducibility analysis were also did for all above fALFF analysis and all results showed high reproducibility (p<0.01, degree of freedom = 27), especially for right fusiform gyrus which demonstrated the highest reproducibility across validation analyses (Information S1).

### Association between morphometric and functional findings

**Figure 3:**
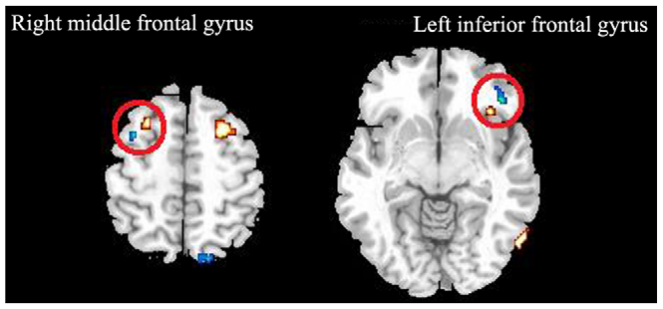
Overlap between the morphometric (red regions) and functional (blue regions) results. Though the decreased ALFF in the right middle frontal gyrus and left inferior frontal gyrus (Within red circle) were found quite near the decreased GMV in the same region, the ALFF in these regions did not correlate with the GMV.

No significant correlation was found between the GMV and ALFF/fALFF in patient group. Though the decreased ALFF in the right middle frontal gyrus and left inferior frontal gyrus were found quite near the decreased GMV in the same region (Figure 3.), there was no overlapping between the ALFF/fALFF and GMV results, and the ALFF/fALFF in these regions did not correlate with the GMV.

## Discussion

The present study examined both anatomical and functional abnormalities in a cohort of drug-naïve MCI patients. Reductions in gray matter volume were observed mainly in the prefrontal, left temporal and posterior cingulate cortex (Figure 1), and volume reduction in the left inferior frontal gyrus was related to the severity of symptom. The regional functional alteration included decreased regional functional mainly in prefrontal, left parietal regions and right fusiform gyrus, while the increased regional functional in limbic and midbrain (Figure 2). However, there is no correlation between morphometric and functional deficits, suggesting an independent alteration of morphometry and function at the transitional state between normal aging and dementia.

Consistent with previous studies [2]–[5], [9], [13], [19], widespread cortical atrophy was found especially in the bilateral frontal lobe, the left temporal gyrus and posterior cingulate cortex. Furthermore, the decreased GMV in the left middle frontal gyrus survived even after family wised error correction (p = 0.047, corrected with FDR). In fact, all these areas are regions typically involved by AD related pathology, as shown by previous MRI [4], [5], [9], [19] and PET [11], [12] studies, and the gray matter atrophy in the prefrontal and posterior cingulate cortex were even thought to predict the cognitive decline in MCI patients as revealed by longitudinal VBM studies [3], [26]. However, we did not find more GMV reduction, especially in the hippocampus which has been the most studied area in MCI cases. In fact, more and more recent studies have shown that structural lesions (eg, neurofibrillary tangles, neuritic plaques) associated with the MCI syndrome are not restricted to hippocampus, but also included the cortical areas [27]. Recent study in patients with mild AD even revealed amyloid deposits in the frontal and parietal lobes instead of the medial temporal lobe structures [28]. Consistent with these findings, our results confirmed the gray matter deficits in the frontal and posterior cingulate cortex in a cohort of drug-naïve MCI patients, suggesting a more diffused cortical deficits than previously thought in MCI.

Furthermore, the decreased volume in the left inferior frontal gyrus was found to be related to the severity of clinical symptom, as measured by the MMSE. The left inferior frontal gyrus has been implicated as an important part in the pathology of MCI, which was thought to be associated with attention and memory processes, including encoding and retrieval and long- and short-term memory [29]–[31]. Increased activation in the left inferior frontal gyrus was associated with improved memory processing in subjects with MCI as revealed in a recent double-blind placebo-controlled fMRI study [32]. However, previous study only has reported positive correlation between the MMSE and the volume of right inferior frontal gyrus [33]. Our results extended previous findings by suggesting that the severity of atrophy in the left inferior frontal gyrus could also be used to evaluate the clinical severity in MCI patients.

Interestingly, resting-state fMRI study revealed both increased and decreased spontaneous activity in MCI group in relative to controls. Abundant previous studies by PET or SPECT have reported hypoactivity in temporal, prefrontal and parietal regions, and decreased glucose metabolism in the temporal and parietal lobes on FDG-PET is recognized as an early imaging marker for the AD pathology [34], [35]. Consistent with the previous study [14], decreased ALFF/fALFF was observed in cingulate cortex, medial prefrontal cortex and some parietal regions; while increased ALFF/fALFF was observed in superior temporal gyrus. Furthermore, the changes of ALFF/fALFF in MCI in the slow-5 band were greater than those in the slow-4. However, we also revealed increased ALFF mainly in limbic regions i.e., parahippocampal gyrus, insula and anterior cingulate cortex (Table 2) and midbrain which had been not been found in the previous study and rarely reported by PET or SPECT studies [11], [12], [34], [35]. In fact, task-fMRI studies in MCI have also reported hyperactivation especially in limbic regions during memory encoding [36]–[38]. One possible explanation for this abnormal hyperactivity is a compensatory neural mechanism, i.e., there is a temporary phase of increased activity in areas like parahippocampus along the course of MCI, which may keep the behavioral performance of MCI patients close to the level of cognitively intact elderly subjects. Around the conversion from MCI to clinical AD, such compensatory ability would be lost, which is then seen as poor task performance. This hypothesis was supported by the finding that greater clinical impairment in MCI subjects as related to smaller hippocampal volumes but greater parahippocampal fMRI activation [36]. Another study even found a negative correlation between hippocampal atrophy and left parahippocampal activation only in MCI instead of in the elderly controls or AD patients [38]. However, we did not observed GMV reduction in hippocampus in drug naïve MCI, suggesting such compensatory overactivity in limbic regions occur before the detectable hippocampal atrophy by VBM. Another possibility is the relative small sample size which would low the statistical power.

Contradictory to our hypothesis, there is no overlap between the morphometric and functional results, or any correlations between the altered regional GMV and ALFF, though the decreased ALFF in the right middle frontal gyrus and left inferior frontal gyrus were found quite near the decreased GMV in the same gyrus (Figure 3). By now, only a few studies used anatomical and functional methods in combination in MCI or AD patients. The results from combination of brain volumetrics and PET [7], [39] showed that hypometabolism largely exceeds gray matter atrophy in most brain regions of AD patients [39]. Another study even observed dissociated effect of atrophy and hypometabolism on episodic memory in MCI patients using the same method [13]. Only two recent studies combine VBM and resting state fMRI in MCI patients, and they also found no overlap between regions with gray matter atrophy and regions with altered functional connectivity [4], [8]. These results altogether with our findings support that brain volume and function may change independently and act different role in the earliest cognitive symptoms of MCI. The altered regional function may represent effects of early neurodegeneration [8].

Several methodological issues should be considered when interpreting the present results. First, although DARTEL-VBM was employed in the current study, which minimizes the contamination of brain tissue with non-brain voxels compared to standard VBM [40] , the use of a Chinese sample may affect the accuracy of normalization because the generic MNI template differs structurally from the brains of non-Caucasian populations [41]. However, as both patients and controls were from a similar ethnic background, the groups differences observed seem unlikely to have been affected by ethnic factors. Second, though we temporally bandpass filtered all fMRI data (0.01–0.08 Hz), and removed components with high correlation to CSF or white matter or with low correlation to gray matter, we cannot completely rule out the influence of physiological noise on our findings due to its variation over time and across subjects. Simultaneous recording of heart rate and respiratory rate and depth during fMRI scanning might help further reduce physiological noise artifacts [42]. Nevertheless, the consistency among the resting state connectivity patterns evidenced by the present data and other studies [23], [43] does reduce the concern about the magnitude of such potential artifacts. Finally, there is a lack of consensus about the exact physiological nature of ALFF. Though ALFF is thought to reflect spontaneous neural activity [19], its exact basis remains to be fully characterized.

Taken together, current study combines anatomical and functional MRI to examine their relationship in a cohort of drug-naïve MCI patients. Findings from the study document that wide spread brain volume reduction accompanied with decreased and increased regional function in MCI, while the anatomical and functional changes were independently. Therefore, the combination of structural and functional MRI methods would provide complementary information and together advance our understanding of the pathophysiology underlying the symptoms of MCI.

## Materials and Methods

### Participants

A total of 35 right-handed subjects were recruited in the present study including 17 drug-naïve MCI and 18 healthy aging controls (Table 3). All patients and healthy controls were recruited at the Mental Health Centre of West China Hospital. This study was approved by the West China Hospital Medical Ethics Committee, and all patients and controls gave written informed consent to their participation. Diagnosis of MCI was determined by consensus of two experienced neurologists according to the criteria for amnestic MCI [44]–[46], which included (a) memory complaint, preferably confirmed by an informant; (b) objective memory impairment, adjusted for age and education; (c) normal or near-normal performance on general cognitive functioning and no or minimum impairment of daily life activities; (d) the Clinical Dementia Rating (CDR) score of 0.5; and (e) not meeting the criteria for dementia according to the DSM-IV (Diagnostic and Statistical Manual of Mental Disorders, 4rd edition, revised). Healthy controls were recruited from the local area by poster advertisement and screened using Structured Interview for DSM-IV Non-Patient Edition to confirm the lifetime absence of psychiatric and neurological illness. Information about age, sex, height, weight, handedness (based on the Annett handedness scale [37]), years of education, duration of illness and clinical symptom ratings were obtained by two experienced clinical neurologists prior to MR examinations. All subjects were evaluated using the Mini Mental State Examination (MMSE), Clock Drawing Test (CDT), Auditory Verbal Learning Test (AVLT), Activity of Daily Living (ADL), Hachinski Ischemic Scaling (HIS), Hamilton Depression Scale (HAMD), and Clinical Dementia Rating Scale (CDR). Age, sex, height, weight and years of education were matched between the MCI and control subjects (Table 3). The following exclusion criteria applied to all subjects: the existence of neurological disorder, alcohol or drug abuse, any physical illness such as hepatitis, brain tumor, or epilepsy as assessed based on clinical evaluations and medical records. Brain MR images (i.e., T1 weighted and T2 weighted images) were inspected by an experienced neuroradiologist, and no gross abnormalities was observed for any subject.

**Table 3:** Demographic information for treatment naïve MCI patients and the healthy controls.

Characteristics	MCI (n = 17)	Controls (n = 18)	p
**Female∶Male**	10∶7	11∶7	1
**Mean age (yrs)**	69.7±7.6 (57–82)	66.5±6.2 (58–82)	0.15
**Education (yrs)**	8.8±4.0 (0–15)	8.4±5.6 (0–17) s	0.79
**Height (cm)**	156.4±7.2 (155–170)	157.2±6.2 (152–170)	0.75
**Weight (kg)**	59.4±6.6 (45–69)	55.8±7.8 (43–75)	0.23
**Illness duration (months)**	47.3±28.5 (3–144)	----	
**CDR**	0.5	0	
**Hachinski**	0	0	
**CDT**	2.3±0.6 (1–3)	2.9±0.3 (2–3)	
**ADL**	21.9±3.2 (20–30)	20±0	0.012
**Hamilton**	3.6±2.7 (0–9)	2.5±2.3 (0–6)	0.62
**MMSE**	25.2±3.5 (17–30)	29.2±0.7 (28–30)	<0.001
**AVLT—immediate recall**	5.8±3.2 (2.2–10.2)	10.1±2.7 (7–15.2)	<0.001
**AVLT—delayed recall**	5.3±4.3 (1–13)	11.9±5.2 (6–15)	<0.001
**AVLT—recognition**	9.3±4.2 (4–12)	12.6±3.8 (6–14)	<0.001

Data are presented as the range of mean±SD (range). MCI: mild cognitive impairment; MMSE: MiniMental State Examination; CDT: Clock Drawing Test; ADL: Activities of Daily Living; HAMD: Hamilton Depression Scale; CDR: Clinical Dementia Rating Scale; AVLT: Auditory Verbal Learning Test.

### Data acquisition

High resolution T1-weighted images were acquired using a 3T MR imaging system (Siemens, Trio) with a volumetric T1 Weighted MPRAGE sequence (TR = 8.5 msec, TE = 3.4 msec, Flip angle = 12°, slice thickness = 1 mm) using an 12 channel phase array head coil. A Field of View (FOV) of 240×240 mm^2^ was used with an acquisition matrix comprising 256 readings of 128 phase encoding steps, producing 156 contiguous coronal slices with slice thickness of 1.0 mm. The final matrix of T1-weighted images was automatically interpolated in-plane to 512×512 which yields an in-plane resolution of 0.47×0.47 mm^2^. MR images sensitized to changes in BOLD signal levels (TR/TE = 2,000/30 ms; flip angle = 90°) were obtained by a gradient-echo echo-planar imaging (EPI) sequence. During the MR examination, subjects were instructed to relax with their eyes closed, keeping awake (confirmed by subjects immediately after the experiment). There were five dummy scans collected before fMRI scans and the first two volumes of fMRI time series were discarded for magnetization stabilization. The slice thickness was 5 mm (no slice gap) with a matrix size of 64×64 and FOV of 240×240 mm^2^, resulting in a voxel size of 3.75×3.75×5 mm^3^. Each brain volume comprised 30 axial slices and each functional run contained 200 image volumes. A traditional T2 weighted image with 20 contiguous axial slices was also acquired to rule out gross brain abnormalities for all subjects.

### VBM analysis

Voxel-based morphometry with DARTEL was performed using SPM8 (Welcome Trust Center for Neuroimaging, London, UK, http://www.filion.ucl.ac.uk/spm/software/spm8/). The procedure included 5 steps [47]: (1) checking for scanner artifacts and gross anatomical abnormalities for each subject; (2) setting the image origin to the anterior commissure; (3) segmenting the images into the GM and WM images in SPM8 toolbox; (4) using the DARTEL toolbox on SPM8 to produce a high-dimensional normalization protocol, following John Ashburner's chapter in its standard version including the Montreal Neurological Institute (MNI) space transformation [48]; (5) The segmented images were modulated with the Jacobian determinants derived from the spatial normalization [40]. Checking for homogeneity across the sample and using standard smoothing by a Gaussian kernel with a full width of half maximum of 8 mm.

### Regional function analysis

Preprocessing and statistical analysis of functional images were carried out using SPM8. For each subject, EPI images were slice-time corrected and realigned to the middle image in the first series, and were subsequently unwraped to correct for susceptibility-by-movement interaction. Individual mean functional images were first coregistered to structural images (3D T1-weighted anatomical images) using a linear transformation, and the parameters were used for coregistration of all the realigned images. All the coregistered images were spatially normalized to the MNI template with the parameters from VBM and each voxel was re-sampled to 3×3×3 mm^3^.

Regional amplitude of low-frequency fluctuations, which are thought to reflect spontaneous neural activity in non-human [20], [49] and human [21], [22] subjects during resting state, was used to evaluate regional functional alteration in MCI. The ALFF was calculated using REST [50] with a procedure similar to that used in our earlier study [14], [51], [52]. In brief, after bandpass filtering (0.01–0.08 Hz) [53] and linear-trend removing, the time series was transformed to the frequency domain using fast Fourier transform (FFT) (parameters: taper percent = 0, FFT length = shortest) and the power spectrum was obtained. Then, the power spectrum obtained by FFT was square root transformed and then averaged across 0.01–0.08 Hz at each voxel. This averaged square root was taken as the ALFF. Fractional ALFF (fALFF) is the fraction of ALFF in a given frequency band to the ALFF over the entire frequency range detectable in the given signal [25]. As fALFF may be more robust against physiological noises, the low-frequency range for BOLD signal were further decomposed into slow 4 [0.027–0.073 Hz] and slow 5 [0.01–0.027 Hz] band [23]. Finally, the fALFF for slow 4 and slow 5 band were calculated. For standardization purposes, the ALFF/fALFF of each voxel was divided by the global mean ALFF/fALFF value [25], [54]. Finally, all the ALFF/fALFF images was smoothed by a Gaussian kernel with a full width of half maximum of 8 mm.

### Statistical Analysis

Voxel-by-voxel based comparisons of gray matter volume (GMV) were performed between groups using two sample t-tests with age, sex and year of education as covariates. The significance of group differences was set at p<0.05 corrected with FDR. To identify the association between structural abnormalities and clinical symptom severity, the average GMV values of all voxels in abnormal areas revealed by VBM were extracted and correlated with Hachinski, CDT, AVLT, MMSE and ADL scores using partial correlation analysis with age, disease duration, sex and year of education as covariates.

ALFF maps in the MCI and control groups were compared on a voxel-wise basis using a two sample t-test in SPM8 with age, sex and year of education as covariates and p<0.05 corrected with FDR was used. Global ALFF between MCI and controls were also compared using a two sample t-test with age, sex and year of education as covariates. To identify the association of regional ALFF with GMV abnormalities, a full voxel-to-voxel correlation of ALFF and GMV images was made using Bivariate correlation analysis in SPM8 and p<0.05 corrected for multiple comparisons was used. To get that, the average ALFF values and the average GMV values of all voxels in abnormal areas revealed by voxel-wise analysis were extracted, respectively. Then the averaged ALFF or GMV values were correlated with the GMV or the ALFF images. To identify the association of regional ALFF with clinical symptom severity, the average ALFF values of all voxels in abnormal areas revealed by voxel-wise analysis were extracted and correlated with the Hachinski, CDT, AVLT, MMSE and ADL scores using partial correlation analysis with age, disease duration, sex and year of education as covariates.

To validate the reproducibility of each region showing changes, leave-one-out cross validation analysis for both structural (VBM) and functional (ALFF/FALFF) analyses was conducted. Specifically, we randomly leave one patient out and performed patient-control comparisons (leading total 17 group comparisons). For each voxel, the ratio of showing significant difference in the measure (i.e., the number of comparisons exhibiting group difference in this voxel divides the total 17 comparisons) was computed as the finding reproducibility of this voxel. The mean reproducibility across all voxels within each region was calculated to measure this region's reproducibility.

## Supporting Information

Information S1 **Includes the methods, results and discussion for fractional ALFF analysis and leave-one-out analysis.** ALFF/fALFF maps for slow 4 [0.027–0.073 Hz] and slow 5 [0.01–0.027 Hz] band in the MCI and control groups were compared on a voxel-wise basis using a two sample t-test in SPM8 with age, sex and year of education as covariates and p<0.05 corrected with FDR was used. Leave-one-out reproducibility analysis were also did for all above ALFF/fALFF analysis and all results showed high reproducibility (p<0.01 FDR corrrected).
(DOC)
