# Supplementary material for: Anatomical and Functional Deficits in Patients with Amnestic Mild Cognitive Impairment
Source: PLoS One. 2012 Feb 3;7(2):e28664. doi: 10.1371/journal.pone.0028664 (PMC3272002; doi:10.1371/journal.pone.0028664)
Supplement: Information S1 — Includes the methods, results and discussion for fractional ALFF analysis and leave-one-out analysis. ALFF/fALFF maps for slow 4 [0.027–0.073 Hz] and slow 5 [0.01–0.027 Hz] band in the MCI and control groups were compared on a voxel-wise basis using a two sample t-test in SPM8 with age, sex and year of education as covariates and p<0.05 corrected with FDR was used. Leave-one-out reproducibility analysis were also did for all above ALFF/fALFF analysis and all results showed high reproducibility (p<0.01 FDR corrrected). (DOC) [file pone.0028664.s001.doc]

Supplementary:

**Methods, Results and Discussion for fractional ALFF and Leave-one-out analysis**

**Methods**

Fractional ALFF (fALFF) is the fraction of ALFF in a given frequency band to the ALFF over the entire frequency range detectable in the given signal[1].The ALFF measures the absolute strength or intensity of LFO, while fALFF represents the relative contribution of LFO within a specific frequency band to the whole detectable frequency range. According to Zuo et al[2]., the full frequency range (0–0.25 Hz) were divided into five different bands: [slow-6 (0–0.01 Hz), slow-5 (0.01–0.027 Hz), slow-4 (0.027–0.073 Hz), slow-3 (0.073–0.198 Hz) and slow-2 (0.198–0.25 Hz)]. The ALFF/fALFF was computed only at the slow-5 and slow-4 bands. The signals of the other band were discarded because they mainly reflect noise instead of physiological signal[2]. Under the studied frequency ranges, ALFF/fALFF of each voxel was computed using REST (State Key Laboratory of Cognitive Neuroscience and Learning in Beijing Normal University; http://resting-fmri.sourceforge.net), and it was further divided by the global mean value to reduce the global effects.

ALFF/fALFF maps for different bands in the MCI and control groups were compared on a voxel-wise basis using a two sample t-test in SPM8 with age, sex and year of education as covariates and p< 0.05 corrected with FDR was used. To identify the association of regional ALFF with GMV abnormalities, the average ALFF values and the average GMV values of all voxels in abnormal areas revealed by voxel-wise analysis were extracted, respectively and were correlated with the GMV or the ALFF images using Bivariate correlation analysis in SPM8 and p< 0.05 corrected for multiple comparisons was used.

**Results**

**ALFF analysis**

ALFF analysis for slow 4 [0.027-0.073 Hz] and slow 5 [0.01-0.027 Hz] band revealed decreased ALFF in cingulate cortex, prefrontal cortex, occipital regions and the bilateral parietal regions, while increased ALFF was observed in superior temporal gyrus, limbic regions and midbrain (Figure S1, S2). Furthermore, the changes of ALFF in MCI in the slow-5 band were greater than those in the slow-4. However, such alteration did not correlated with any of the clinical symptom severity.

Global ALFF and ALFF for slow 4 and slow 5 between MCI and controls were not significant (p > 0.05).

**fALFF study**

We compared the fALFF between MCI and controls and found that the MCI group showed wide spread decreased fALFF mainly in bilateral medial/dorsallateral prefrontal cortex, cingulate gyrus, occipital regions and left cerebellum. Increased fALFF was revealed only in brain stem regions. Compared with ALFF findings, fALFF revealed more regions with decreased activity, while less regions with increased activity. fALFF analysis for slow 4 [0.027-0.073 Hz] and slow 5 [0.01-0.027 Hz] band also revealed decreased fALFF in cingulate cortex, prefrontal cortex, occipital regions and the bilateral parietal regions, while increased fALFF was observed in superior temporal gyrus, limbic regions and midbrain (Figure S1, S2). Furthermore, the changes of fALFF in MCI in the slow-5 band were greater than those in the slow-4. However, no overlap nor inter-subject correlation was observed between the fALFF and GMV alternations. Global fALFF and fALFF for slow 4 and slow 5 between MCI and controls were not significant (p > 0.05).

**Leave-one-out analysis**

Leave-one-out reproducibility analysis were also did for all above ALFF/fALFF analysis and all results showed high reproducibility (p < 0.01 FDR corrrected). Results were shown in Figure S3-S5.

**Discussion**

Consistent with the previous study[3], decreased ALFF/fALFF was observed in cingulate cortex, medial prefrontal cortex and some parietal regions; while increased ALFF/fALFF was observed in superior temporal gyrus. Furthermore, the changes of ALFF/fALFF in MCI in the slow-5 band were greater than those in the slow-4 (Figure S1, S2). However, we also revealed increased ALFF mainly in limbic regions and midbrain which had been not been found in our previous study. In fact, task-fMRI studies in MCI have also reported hyperactivation especially in limbic regions during memory encoding [4-6]. One possible explanation for this abnormal hyperactivity is a compensatory neural mechanism, i.e., there is a temporary phase of increased activity in areas like parahippocampus along the course of MCI, which may keep the behavioural performance of MCI patients close to the level of cognitively intact elderly subjects. Around the conversion from MCI to clinical AD, such compensatory ability would be lost, which is then seen as poor task performance. This hypothesis was supported by the finding that greater clinical impairment in MCI subjects as related to smaller hippocampal volumes but greater parahippocampal fMRI activation [4]. Another possibility is the relative small sample size which would low the statistical power. However, no overlap nor inter-subject correlation was observed between the ALFF/fALFF and GMV alternations.


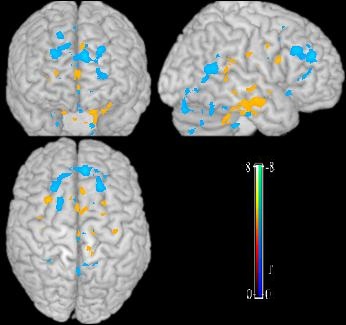

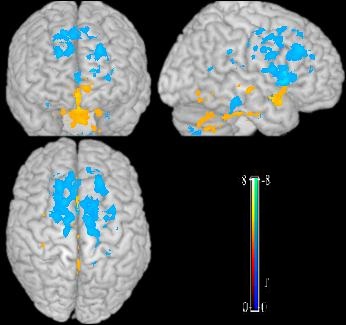


0.027ALFF 0.027fALFF

Figure S1. Group comparison of ALFF/fALFF in the slow-5 band between MCI and Controls. Blue regions represent decreased ALFF/fALFF in MCI, while yellow regions mean increased ALFF/fALFF. Statistical significant were set at P < 0.05 corrected with FDR.


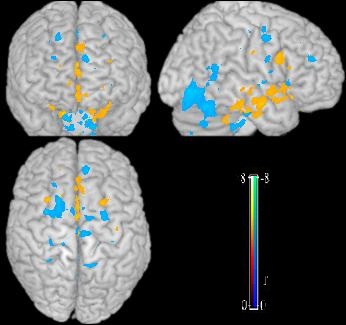

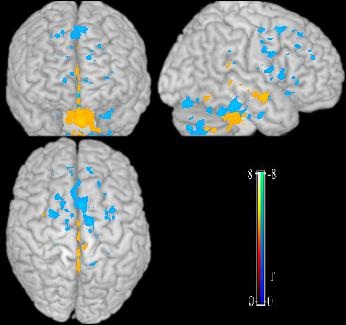


0.073ALFF 0.073fALFF

Figure S2. Group comparison of ALFF/fALFF in the slow-4 band between MCI and Controls. Blue regions represent decreased ALFF/fALFF in MCI, while yellow regions mean increased ALFF/fALFF. Statistical significant were set at P < 0.05 corrected with FDR.


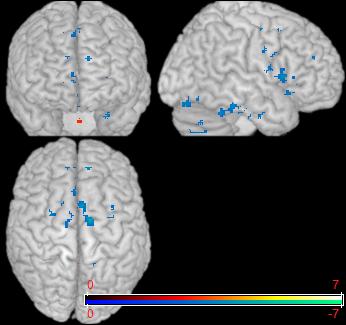

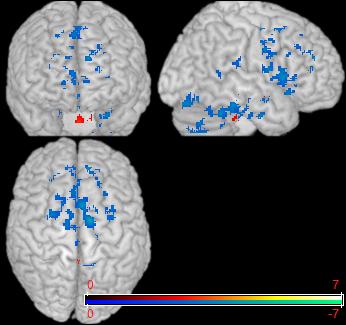


ALFF fALFF

Figure S3. Results of one sample t test for leave-one-out evaluation on ALFF/fALFF. The blue and red regions represent regions of decreased/increased ALFF/fALFF with significant high reproducibility (t> 3.4, P < 0.05 corrected with FDR.


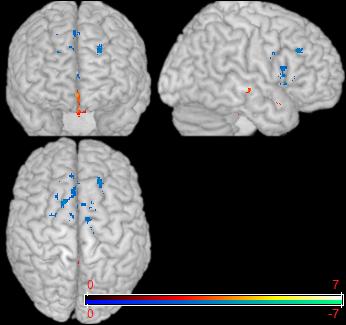

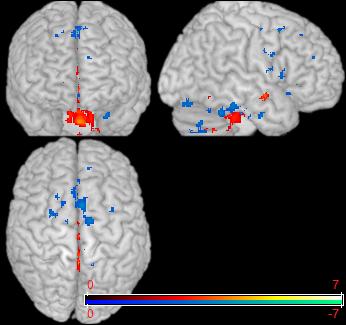


0.027ALFF 0.027fALFF

Figure S4. Results of one sample t test for leave-one-out evaluation on ALFF/fALFF in the slow-5 band. The blue and red regions represent regions of decreased/increased ALFF/fALFF with significant high reproducibility (t> 3.4, P < 0.05 corrected with FDR.


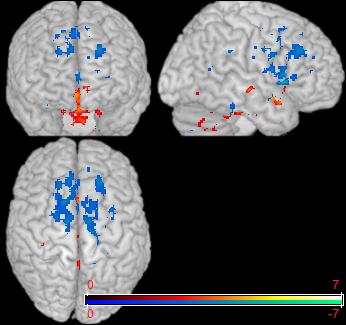

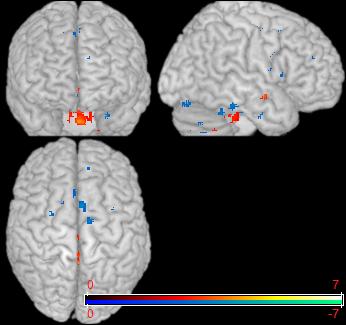


0.073ALFF 0.073fALFF

Figure S5. Results of one sample t test for leave-one-out evaluation on ALFF/fALFF in the slow-4 band. The blue and red regions represent regions of decreased/increased ALFF/fALFF with significant high reproducibility (t> 3.4, P < 0.05 corrected with FDR.

**References**

1. Zou QH, Zhu CZ, Yang Y, Zuo XN, Long XY, et al. (2008) An improved approach to detection of amplitude of low-frequency fluctuation (ALFF) for resting-state fMRI: fractional ALFF. J Neurosci Methods 172: 137-141.

2. Zuo XN, Di Martino A, Kelly C, Shehzad ZE, Gee DG, et al. (2010) The oscillating brain: complex and reliable. Neuroimage 49: 1432-1445.

3. Han Y, Wang J, Zhao Z, Min B, Lu J, et al. (2011) Frequency-dependent changes in the amplitude of low-frequency fluctuations in amnestic mild cognitive impairment: a resting-state fMRI study. Neuroimage 55: 287-295.

4. Dickerson BC, Salat DH, Bates JF, Atiya M, Killiany RJ, et al. (2004) Medial temporal lobe function and structure in mild cognitive impairment. Ann Neurol 56: 27-35.

5. Dickerson BC, Salat DH, Greve DN, Chua EF, Rand-Giovannetti E, et al. (2005) Increased hippocampal activation in mild cognitive impairment compared to normal aging and AD. Neurology 65: 404-411.

6. Hamalainen A, Pihlajamaki M, Tanila H, Hanninen T, Niskanen E, et al. (2007) Increased fMRI responses during encoding in mild cognitive impairment. Neurobiol Aging 28: 1889-1903.
